# Non-esterified fatty acids in the ovary: friends or foes?

**DOI:** 10.1186/s12958-020-00617-9

**Published:** 2020-06-06

**Authors:** Vijay Simha Baddela, Arpna Sharma, Jens Vanselow

**Affiliations:** Institute of Reproductive Biology, Leibniz Institute for Farm Animal Biology (FBN), Wilhelm-Stahl-Allee 2, 18196 Dummerstorf, Germany

**Keywords:** NEFAs, Granulosa cells, Oocyte, Ovary, Metabolic diseases

## Abstract

A majority of common metabolic diseases can result in excessive lipolysis, leading to elevated levels of non-esterified fatty acids (NEFAs) in the body fluids. In females, increased NEFA levels in the follicular fluid markedly alter the functions of intrafollicular cells such as granulosa cells (GCs) and oocytes. Therefore, elevated levels of NEFAs have been suggested to be a significant player of subfertility in females of both human and economically important animal species such as cattle, buffalo, sheep, pig, chicken, and dog. However, the effects imposed by saturated and unsaturated fatty acids (SFAs and UFAs) on ovarian follicles are controversial. The present review emphasizes that SFAs induce apoptosis in granulosa and cumulus cells of ovarian follicles in different species. They further could adversely affect oocyte maturation and developmental competence. Many types of UFAs affect steroidogenesis and proliferation processes and could be detrimental for follicular cells, especially when at elevated concentrations. Interestingly, monounsaturated fatty acids (MUFAs) appear to contribute to the etiology of the polycystic ovarian syndrome (PCOS) as they were found to induce the transcription and translation of the androgenic transcription factor SOX9 while downregulating its estrogenic counterpart FOXL2 in GCs. Overall, this review presents our revised understanding of the effects of different fatty acids on the female reproductive success, which may allow other researchers and clinicians to investigate the mechanisms for treating metabolic stress-induced female infertility.

## Introduction to the follicular physiology

**Fig. 1:**
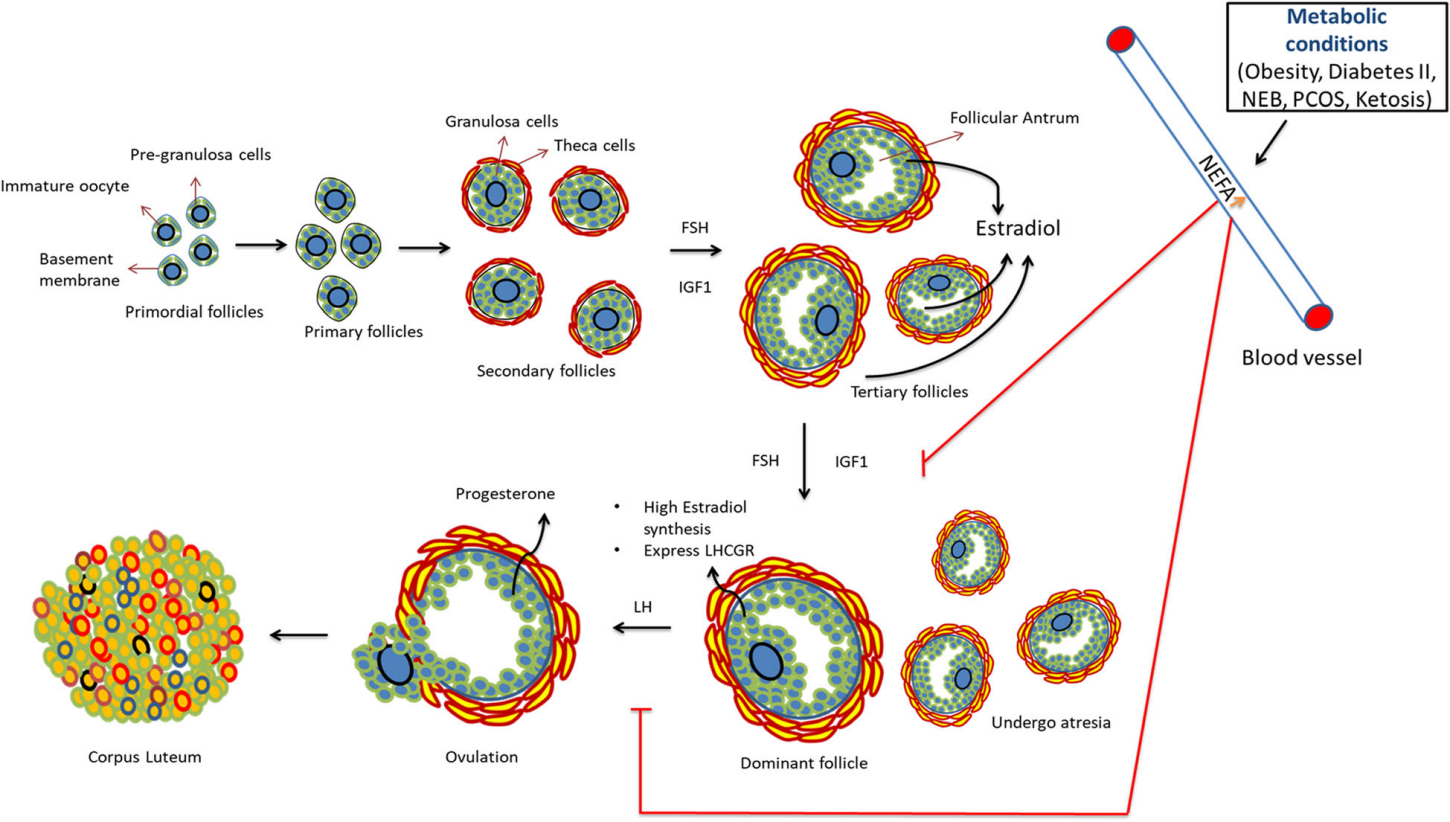
Folliculogenesis: Ovarian follicles undergo a sequential development in order to release a mature oocyte for fertilization. Elevated levels of NEFAs affect the ovarian follicular development by preventing dominant follicle formation and inhibiting ovulation

Ovarian follicles are a large but limited pool of complex miniature structures in the ovary. They are originally assembled in the form of primordial follicles containing a single layer of flattened pre-granulosa cells surrounding a dormant immature oocyte. Each primordial follicle is enclosed by a basement membrane, which separates the follicle from the rest of the ovarian stroma throughout follicular development. Some of the primordial follicles commence growth as soon as they are formed and develop into primary and some eventually into secondary follicles containing distinct cuboidal shaped granulosa cells (GCs) with a larger oocyte in the center. However, a majority of primordial follicles stay in a quiescent stage for months or years before commencing further development [1]. Follicles with a single layer of GCs are called primary follicles, and those with multiple GCs layers are called secondary follicles. The development of primordial follicles into secondary follicles takes place in a gonadotropin independent manner. However, expression of the gonadotropin receptor, i.e., follicle-stimulating hormone receptor (FSHR) can already be detected in the GC layer of primary follicles [2]. Therefore, the development from primary to secondary follicles is argued to be a gonadotropin sensitive mechanism. In secondary follicles, additional layers such as the theca interna and theca externa start appearing on the outer side of the basement membrane and contribute to follicular development [3]. The developmental events from secondary follicles into preovulatory follicles take place in a gonadotropin dependent manner as the GCs of ovarian follicles can be stimulated by follicle stimulating hormone (FSH). Together with hepatic insulin-like growth factor 1 (IGF-1), FSH induces proliferation and steroidogenesis in GCs and supports the formation of the follicular antrum [4]. Antral ovarian follicles are also called tertiary follicles and their fate is largely determined by the function of GCs. A pool of small antral follicles are recruited into the ovarian cycle in every menstrual or estrous cycle. In response to FSH and IGF-1, one of the recruited antral follicles becomes the dominant follicle and produces ample amounts of the female sex hormone 17β-estradiol (E2), which in turn induces the release from the anterior pituitary of the second gonadotropin hormone i.e. luteinizing hormone (LH). The antral follicle that has achieved optimal growth and is present at the precise time window during the follicular phase of the ovarian cycle survives as a dominant follicle in each ovarian cycle. The pre-ovulatory LH surge triggers the release of the mature oocyte from the fully developed dominant follicle for fertilization. The somatic cells of the theca and granulosa layers from the ovulated follicle subsequently differentiate into the corpus luteum (CL) (Fig. 1). In mono-ovulatory animals such as humans and bovines, only one of the recruited follicles will be selected to become a dominant follicle while others undergo atresia. In contrast, in poly-ovulatory animals such as pigs, dogs, and rodents, multiple dominant follicles are selected to undergo ovulation per ovarian cycle.

In addition to timely endocrine stimulations, follicular development depends on the health and energy status of the animal. It has been shown that folliculogenesis is dramatically affected during periods of limited food availability (e.g., malnutrition or fasting) or increased energy demands (e.g., during lactation and negative energy balance (NEB)), which are not met by compensatory food intake [5–7]. Interestingly, over-nutrition associated metabolic diseases, such as obesity and diabetes 2, could also impair the development of ovarian follicles in many different species, including humans, mice, and rats [8, 9]. At least one biochemical factor that has been commonly identified as a characteristic marker for both under- and over-nutrition metabolic conditions is the elevated levels of non-esterified fatty acids (NEFAs) in the circulation. Significantly higher serum concentrations of NEFAs were found in heifers after short term fasting [7] and in dairy cows during negative energy balance [10, 11], as well as in obese women [12]. Furthermore, some reports show that polycystic ovarian syndrome (PCOS), which is argued to be a metabolic disease, is also characterized by the presence of excess amounts of NEFA in body fluids [13–15]. However, a significant increase of NEFA concentrations was not consistently found [13, 16], indicating that these diseases are not always associated with elevated NEFA levels (Tables 1 and 2). This might be possibly due to differential genetic and environmental factors and needs future investigation.

**Table 1:** Concentrations of NEFAs in different metabolic diseases in humans

Species	Condition	Biofluid	Concentration of NEFA	Ref.
Women	Obese non-diabetic	Plasma	290 μM	[24]
	Obese type 2 Diabetes	Plasma	621 μM ^a^	
Human (sex not defined)	Healthy	Plasma	0.18 ± 0.09 (SD) g/L	[25]
	Type 2 Diabetes	Plasma	0.45 ± 0.21 (SD) g/L	
Women	Normal weight	Follicular Fluid	0.22 ± 0.02 (SD) mM	[12]
	Overweight	Follicular Fluid	0.24 ± 0.03 (SD) mM	
	Obese	Follicular Fluid	0.31 ± 0.08 (SD) mM ^a^	
Women	18.5 ≤ BMI ≤24.9 (*n* = 60)	Serum	0.60 ± 0.20 (SD) mM	[16]
	25.0 ≤ BMI ≤29.9 (*n* = 26)	Serum	0.70 ± 0.23 (SD) mM	
	BMI ≥30.0 (*n* = 20)	Serum	0.70 ± 0.20 (SD) mM	
Women	Non Obese	Follicular Fluid	0.16 ± 0.02 (SEM) meq/L	[26]
	Obese	Follicular Fluid	0.38 ± 0.04 (SEM) meq/L ^a^	
Women	Non Obese	Plasma	0.49 ± 0.22 (SD) mM	[13]
	Obese	Plasma	0.37 ± 0.18 (SD) mM	
	PCOS Non Obese	Plasma	0.49 ± 0.23 (SD) mM	
	PCOS Obese	Plasma	0.70 ± 0.13 (SD) mM ^a^	
Women	Healthy Control	Serum	4.36 ± 2.52 (SD)mg/dl	[15]
	PCOS	Serum	6.93 ± 3.51(SD) mg/dl ^a^	
Women	Healthy fertile	Serum	3.1 ± 0.01 mg/dl	[27]
	Infertile	Serum	4.7 ± 0.08 mg/dl ^a^	
Women	Healthy fertile	Follicular Fluid	3.5 ± 0.03 mg/dl	[27]
	Infertile	Follicular Fluid	6.0 ± 0.1 ^a^ mg/dl	

^a^, significantly different levels as indicated by authors; *SD* standard deviation, *SEM* standard error of the mean

**Table 2:** Concentrations of NEFAs in different metabolic diseases in animals

Cows	7 days pre-parturition	Serum	~ 0.2 mM	[17]
	16 days post- parturition	Serum	0.4–1.2 mM ^a^	
	44 days post-parturition	Serum	0.1–0.3 mM	
Cows	16 days post- parturition	Follicular Fluid	0.2–0.6 mM	[17]
	44 days post-parturition	Follicular Fluid	0.1–0.3 mM ^a^	
Cows	Control	Follicular Fluid	control level	[7]
	Fasting (4 days)	Follicular Fluid	higher level ^a^	
Cows	Control	Serum	control level	[7]
	Fasting (4 days)	Serum	higher level ^a^	
Cows	Cycling	Plasma	0.21 ± 0.05 mM	[18]
	Inactive ovary	Plasma	0.32 ± 0.12 mM ^a^	
Cows	Cycling cows	Blood	0.4 ± 0.1 (SEM) mM ^a^	[19]
	Cystic ovarian cows	Blood	0.7 ± 0.1 (SEM) mM	
Ewes Pregnant	Control	Serum	0.65 mM	[20]
	Subclinical ketosis	Serum	1.02 mM ^a^	
Ewes Lambed	Control	Serum	0.47 mM	
	Subclinical ketosis	Serum	0.69 mM ^a^	
Ewes Lactating	Control	Serum	0.21 mM	
	Subclinical ketosis	Serum	0.45 ± 0.03 (SD) mM ^a^	
Dog	Lean dog	Plasma	0.97 ± 0.09 (SEM) mM	[21]
	Obese dog	Plasma	1.59 ± 0.12 (SEM) mM	

^a^, significantly different levels as indicated by authors; SD, standard deviation; SEM, standard error of the mean

## Fatty acids

Fatty acids (FAs) are carboxylic acids with an aliphatic chain of different lengths and saturation levels. FAs are broadly classified into two categories: 1) saturated fatty acids (SFAs) and 2) unsaturated fatty acids (UFAs). SFAs contain only single bonds between the carbons of their aliphatic chain, e.g. palmitic acid (PA, 16:0) and stearic acid (SA 18:0), whereas UFAs contain one or more double bonds. e.g., oleic acid (OA 18:1) and linoleic acid (LA 18:2) [22]. Furthermore, UFAs can be classified into 2 subcategories: i) monounsaturated fatty acids (MUFAs), which contain only one double bond, e.g., palmitoleic acid (16:1) and OA (18:1), and ii) polyunsaturated fatty acids (PUFAs), containing two or more double bonds in the aliphatic chain. UFAs are also classified on the basis of the position of the first double bond starting from the methyl end of the carbon chain. Omega-3 FAs have the first double bond at the third carbon atom and include alpha-linolenic acid (ALA 18:3 n-3), eicosapentaenoic acid (EPA 20:5 n-3), and docosahexaenoic acid (DHA 22:6 n-3). Omega-6 FAs have the first double bond at the sixth carbon atom, which produces LA and its derivative arachidonic acid (AA 20:4 n-6). Omega-9 FAs have the first double bond at the ninth carbon atom (e.g., OA). The ω-6 and ω-3 FAs such as LA and ALA are called essential fatty acids (EFA) as humans cannot synthesize them de novo. LA is the parent FA for the remaining ω-6 EFAs, whereas ALA is the parent FA for the remaining ω-3 EFAs. Therefore, humans need dietary supplementation with LA and ALA to produce higher-order UFAs such as AA and docosahexanoic acid (C22:6).

FAs are essential constituents of all living cells and have significant roles as components of biomembranes, cell signaling (steroid hormones and prostaglandins), and energy substrates (e.g., in the form of di- or tri- acylglycerols). They are widely favored as the preferred form of stored energy because of their low hydrodynamic diameter and the incredibly high amount of energy released upon their oxidation compared to carbohydrates. During periods of starvation/fasting, de-esterification of FAs from stored lipids of the adipose tissue takes place by the action of a hormone-sensitive lipase, resulting in the temporary elevation of NEFAs in the circulation for coping with the body’s energy demands [7, 23]. However, such lipolysis is repressed in healthy animals by the action of insulin, whose levels are increased after an energy-rich meal.

Severe and unregulated lipolysis is a hallmark of various metabolic diseases such as obesity, diabetes 2, NEB, and subclinical ketosis and it causes continuously elevated levels of NEFAs in the body fluids of humans and animals (Table 1 and Table 2) [7, 11, 17]. Elevated levels of NEFAs in the circulation, in turn, enter the follicular fluid and alter the concentrations in developing ovarian follicles [28]. Valckx et al. 2014 [29] showed that in vitro exposure of murine ovarian follicles to elevated levels of NEFAs resulted in the impairment of ovarian steroidogenesis and oocyte competence for fertilization. To further understand the physiological responses under in situ conditions, Sharma et al. 2019 [30] took advantage of the ultrasound-guided injection approach to inject NEFAs (oleic acid; C18:1) into the dominant follicles of heifers in situ. Interestingly, the NEFA injected animals showed reduced ovulation rates and reduced production of E2 hormone compared to that of the control group of animals (Fig. 1). Therefore, it can be implied that the ovarian dysfunction in animals/humans observed during metabolic diseases/conditions can be due to elevated levels of accumulating follicular NEFAs. In the follicular fluid, PA, SA, and OA together contribute up to 45% of the total NEFA concentration. These fatty acid levels can further increase or even double in the follicular fluid during specific metabolic conditions in humans and animals [7, 12, 17]. Therefore, a major emphasis will be given to the effects of these FAs in the following sections.

## Effects of NEFAs on GC function

**Fig. 2:**
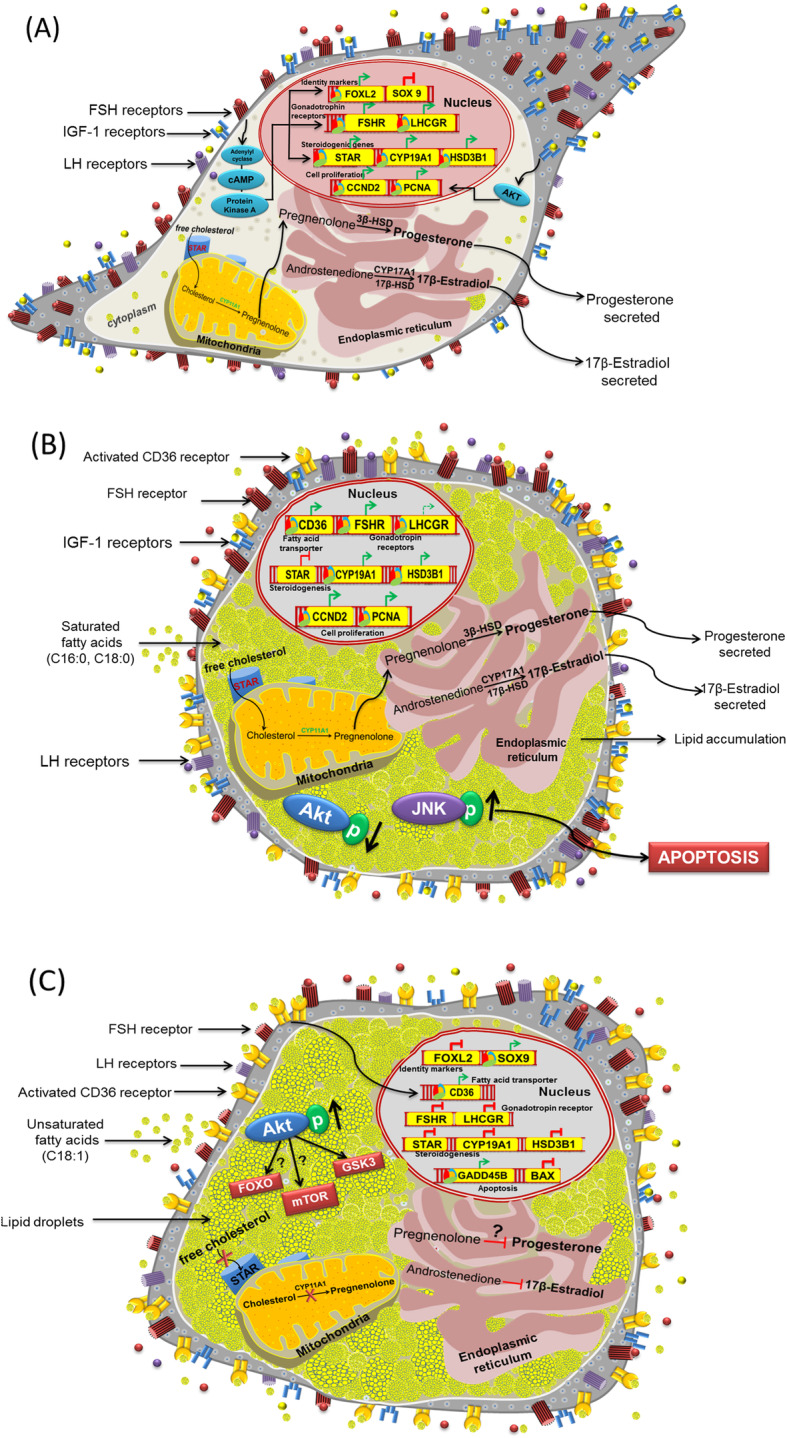
Effect of FAs on the physiology of cultured GCs: **a** Under standard in vitro culture conditions, GCs show a typical fibroblast-like morphology in the presence of FSH and IGF1. GCs display an active expression of gonadotrophin (FSHR and LHCGR) and IGF-1 receptors whose signaling could promote steroidogenesis (*CYP19A1*, *STAR*, and *HSD3B1)* and cell proliferation (CCND2 and PCNA) via PKA (protein kinase A) and Akt activation. **b** Saturated fatty acids (C16:0 and C18:0) induce adverse morphological changes in GCs with an increasing number of cells undergoing apoptosis. Decreased phosphorylation of Akt was reported in GCs. Increased expression of CD36 (fatty acid transporter), IGF-1 and FSH regulated genes can be found in GCs (**c**) Unsaturated fatty acids, such as OA, at elevated concentrations, also induce adverse morphological changes with increased expression of *CD36* (fatty acid transporter) leading to lipid accumulation. Increased Akt phosphorylation is hypothesized to occur upon OA treatment. The expression of gonadotrophin receptors, steroidogenic and proliferation genes is down-regulated

GCs are steroidogenic somatic cells of the ovarian follicle. They play an indispensable role in the nourishment of the oocyte and regulation of the ovarian cycle. Under healthy conditions, GCs of dominant follicles synthesize and secrete ample amounts of E2 upon stimulation with FSH and IGF-1 (Fig. 2). The increased systemic levels of E2 trigger the release of LH from the anterior pituitary in a positive feedback mechanism via gonadotropin-releasing hormone (GnRH) and induce ovulation. Therefore, compounds that affect the function of GCs could potentially negatively impact female fertility.

FAs have been reported to alter GC function by affecting steroidogenesis, proliferation, and apoptotic processes necessary for follicular development [26, 30, 31]. Elis et al. 2015 [32] have shown the mechanisms by which the FA metabolism is linked to GC function in bovines using chemical inhibitors such as etomoxir and C75 (4-methylene-2-octyl-5-oxotetra- hydrofuran3-carboxylic acid). Etomoxir prevents FA oxidation by irreversibly inhibiting the carnitine palmitoyltransferase-1 enzyme. C75 is an inhibitor of the fatty acid synthase complex, thus preventing fatty acid biosynthesis. Both inhibitors decreased the IGF-1 induced proliferation of GCs and affected the phosphorylation of key enzymes of the cellular metabolism such as AMPK (5′ adenosine monophosphate-activated protein kinase) and acetyl CoA carboxylase. These data suggest that basal FA metabolism (anabolism and catabolism) is essential for GC function and optimal follicular growth.

A growing number of studies describe that an excess amount of NEFAs disrupts the function of GCs during metabolic diseases. One of the apparent effects of elevated NEFA levels is the induction of dramatic morphological alterations in GCs. In addition to the significant impact on the expression of critical genes and hormone production, Yenuganti et al. 2016 [33] and Sharma et al. 2019 [30] reported the formation of foam cell-like structures in cultured bovine GCs upon treatment with increased levels of NEFAs such as OA, PA, and SA; the formation was possibly the result of excessive lipid droplet accumulation. The altered morphology of GCs has been attributed to the active uptake of FAs by GCs via the CD36 translocase system. CD36 is an 88-kDa transmembrane glycoprotein with lipid-based ligand binding for low-density lipoproteins (LDL), high-density lipoproteins (HDL), very low-density lipoproteins (VLDL), fibrillar β-amyloid, and collagen molecules. Expression of CD36 induces the accumulation of conjugated linoleic acids *(*CLA) in human macrophages and 15P-1 cell lines (testicular cells) of mice [34, 35]. Similarly, CD36 protein expression is increased upon feeding ALA enriched diets to rats promoting the transport of lipids into their resting skeletal muscles [36]. This information suggests that CD36 is actively involved in the uptake of FAs in mammalian cells. Upon cellular uptake, different FAs appeared to elicit different responses, depending on the degree of saturation/unsaturation of FAs.

### Saturated fatty acids

Accumulating evidence indicates that SFAs have adverse effects on GC function. PA and SA have been found to induce apoptosis in cultured human primary GCs and in human KGN cells in a dose-dependent manner [37]. Particularly, at a concentration of 300 μM, PA was found to cause a significant decline in the expression of the anti-apoptotic protein BCL2 (B-cell lymphoma 2) and an increase in the expression of BAX (BCL2 associated X protein), a pro-apoptotic protein, in human primary GCs. Distinct DNA fragmentation in PA (300 μM) treated GCs confirmed the apoptotic effects of SFAs. However, PA had no apoptotic effects at a concentration of 100 μM, whereas SA could still pose a marginal yet significant decrease in cell viability at 100 μM, which became very severe as the concentration of SFAs was increased. This indicates that elevated concentrations (> 100 μM) of SFAs are cytotoxic to human GCs. Similar negative effects of SFAs have been reported in mice, cows, and pigs [30, 38]. Shibahara et al. 2020 [39] have recently reconfirmed the proapoptotic effects of SFAs as they observed induced expression of caspase-3 and C/EBP homologous *protein* (CHOP) and decreasing Akt phosphorylation in porcine GCs. These effects were observed in association with reduced cell viability and increased ceramide accumulation.

FAs are well-known ligands for peroxisomal proliferator-activated receptors (PPARs) in mammalian cells. In an interesting experiment, Mu et al. 2001 [37] induced PPAR signaling with a synthetic FA analog, fenofibrate. The results revealed that fenofibrate could not induce apoptosis in human GCs, suggesting that FA induced apoptosis may not be the result of direct interactions of FAs with PPAR in GCs. Interestingly, cellular apoptosis seems to be mediated by metabolites of SFAs as shown in chicken and human GCs. PA-induced apoptosis of GCs is accompanied by increased expression of genes coding for various enzymes of the FA and lipid metabolism, including carnitine palmitoyl transferase-1 (CPT1), serine palmitoyltransferase (SPT), acyl CoA oxidase (ACO), and sphingomyelinase (SMASE) [40]. Therefore, the effects of different chemical inhibitors that could inhibit these enzymes were tested to identify the mechanisms involved in FA induced cell death in GCs. Surprisingly, independent supplementation with triacsin C (fatty acyl CoA synthase inhibitor), imipramine (sphingomyalinase inhibitor), fumonisin B1 (ceramide synthesis inhibitor) and pyrrolidine dithiocarbamate (free radicle scavenger) rescued PA-induced cell death in chicken GCs [40]. Similarly, triacsin-C supplementation was found to significantly decrease both PA and SA induced cell death in human GCs [37]. However, treatment with fumonisin B1 and the nitric oxide synthase inhibitor aminoguanidine could not inhibit PA-induced apoptosis in human GCs. These above studies suggest that especially acylated forms of FAs, which are precursor forms of FA oxidation, are detrimental for cell health. Further validation of this phenomenon was provided when human GCs were treated with acyl derivates of different SFAs and UFAs. Similar to the effects of PA and SA, palmitoyl CoA and stearoyl CoA significantly decreased cell viability in a dose-dependent manner [37]. All these observations indicate that excessive β-oxidation of SFAs could be a potential inducer of apoptosis and cell death, possibly by generating an excessive amount of ROS in GCs of different species.

However, PA and SA could not elicit early apoptotic effects as shown by an Annexin V assay in bovine GCs which might be due to increased transcription of FSH signaling induced genes such as cytochrome P450 family 19 subfamily A member 1 (*CYP19A1*), follicle stimulating hormone receptor (*FSHR*), luteinizing hormone/choriogonadotropin receptor (*LHCGR)* as well as enhanced synthesis of E2 by PA and SA [6, 30]. It has been shown that higher E2 levels protect the cells from Fas ligand-mediated apoptosis and induce proliferation by increasing the percentage of cells entering the S phase of the cell cycle [41]. Similar upregulation of steroidogenesis has been reported in bovine adrenal cells exposed to SFAs [42]. However, Vanholder et al. 2005 [6] showed in bovine GCs that both PA and SA could significantly increase the number of dead cells under in vitro conditions. These contradictory observations can be very likely explained by the opposing effects of SFAs and E2 on apoptosis. On the one hand, apoptosis can be promoted by PA and SA treatment, but on the other hand, these detrimental effects of SFAs can be overridden by increased E2 production in E2 active GC. Therefore, the E2 active state of GCs must be acknowledged while discussing the effects of FA.

SFAs were also found to have detrimental effects on bovine cumulus cells, which are distinct from mural GCs and have a relatively lower steroidogenic ability. In vitro exposure of bovine cumulus-oocyte complexes (COCs) to PA and SA induces endoplasmic reticulum (ER) stress, mitochondrial damage and apoptosis in cumulus cells [43, 44]. ER stress in cumulus cells has been determined by measuring the gene expression of *ATF4* (activating transcription factor 4) and *HSPA5* (heat shock protein family A (Hsp70) member 5) [43]. The expression of *ATF4* and *HSPA5* genes was also up-regulated in mouse COCs exposed to the lipid-rich follicular fluid compared to lipid-deprived follicular fluid [26].

IGF-1 signaling plays a key role in GC steroidogenesis and proliferation [45]. More recently, it was observed that PA inhibits glucose uptake and induces insulin resistance in KGN cells by inhibiting IGF-1 induced Akt phosphorylation while increasing c-Jun N-terminal kinase (JNK) phosphorylation [46] (Fig. 2). Furthermore, the inhibition of JNK phosphorylation reversed the PA-induced downregulation of Akt phosphorylation. These results are in line with earlier observations made by Walsh et al. 2012 [47] that NEB like conditions such as acute dietary restriction, which elevate NEFA levels, could affect the IGF-1 and gonadotropin signaling associated gene expression in GCs, leading to an anovulatory phenotype in animals.

Overall, it is apparent that the metabolites of SFAs could induce apoptosis in GCs of different species, which may be ameliorated by inhibitors of FA metabolism and antioxidant molecules. In any case, the E2 active phenotype of GCs may play a key role in the apoptotic response of GCs to elevated SFAs.

### Unsaturated fatty acids

Similar to SFAs, UFAs are also known inducers of fatty acid transporters, *CD36,* and solute carrier family 27 member 1 in GCs, thus promoting their active uptake [33]. However, in contrast, UFAs show mostly opposite biological effects on GC function compared to SFAs. It has been shown that UFAs can counteract the cytotoxic effects of SFAs by stimulating triacyl glyceride formation, which leads to a reduction of SFA concentrations available for oxidative processes, the byproducts of which are primarily cytotoxic [40, 48]. However, MUFAs such as OA have been shown to cause a significant decline in the proliferation of in vitro cultured GCs of both human and bovine origin [6, 28, 37]. On the other hand, Sharma et al. 2019 [30] reported upregulation of the proliferation marker cyclin D2 (CCND2) in bovine GCs by SFAs such as PA and SA. These effects clearly suggest that different, and perhaps opposite signaling mechanisms are influenced by SFAs and UFAs. This can be partially explained by data from rat skeletal muscle cells [49], which showed that OA could induce Akt phosphorylation and also reverse the PA-induced dephosphorylation of Akt by activating the PI3K (phosphoinositide 3-kinase-3-kinase) pathway in skeletal muscle cells. However, the downstream pathways of Akt such as forkhead box protein O1 (FOXO1), *glycogen synthase kinase 3* (GSK3) and mammalian target of rapamycin (mTOR), which could hold the key to explaining these observed effects, have not yet been studied with respect to UFAs.

OA and LA have been found to elicit only a marginal apoptotic response at a concentration of 300 μM, but unlike SFAs, they have no effect at lower concentrations. AA (arachidonic acid; 20:4) could not induce any apoptotic response within its physiological range (1 to 10 μM) in human GCs [37]. Furthermore, Valckx et al. 2014 [12] revealed that OA could reduce the expression of *BAX* and induce *BCL2* and *Gadd45b* (growth arrest and DNA damage-inducible beta) expression in human GCs. A recent report revealed that AA could promote the survival of bovine GCs at lower concentrations (50 μM) but had detrimental effects at higher concentrations (200 μM).

OA downregulates the mRNA expression of different genes associated with FSH and LH signaling in bovine GCs such as steroidogenic acute regulatory protein (*STAR*), *CYP19A1, FSHR, LHCGR,* cytochrome P450 family 11 sub-family A member 1 (*CYP11A1*), hydroxy-delta-5-steroid dehydrogenase, 3 beta- and steroid delta-isomerase 1 (*HSD3B1*), *CCND2* and proliferating cell nuclear antigen (*PCNA*), which are induced by SFAs [30]. Estrogen receptor 2 (*ESR2*), follistatin (*FST*), and *PPARG* are among the other genes that were down-regulated by OA in these cells [50]. However, the protein abundance of the above genes has yet to be determined. Similar to OA, CLA, an isomer of LA has been found to affect FSH and IGF-1 induced steroidogenesis by down-regulating the transcription of *CYP19A1*, insulin-like growth factor 1 receptor (*IGFR1*) and *GATA4* genes [51]. These observed effects were attributed to decreased levels of Akt phosphorylation and increased protein abundance of PPARG and phosphatase and tensin homolog (PTEN) by CLA in buffalo GCs [51]. It was reported that PPARG activation decreased E2 production by inducing the ubiquitination of cyclin D1 and estrogen receptor α, and that it inhibited the expression of *CYP19A1* through nuclear factor-kappaB (NFkB) activation [52, 53]. Nevertheless, decreased E2 levels by both CLA and OA suggest that UFAs are detrimental to female steroid production during metabolic stress conditions. However, the opposite regulatory effects on Akt phosphorylation by OA and CLA is very intriguing for future studies to understand the importance of Akt signaling concerning different FAs.

It seems that UFAs play an important role in PCOS pathophysiology, whose etiology is largely attributed to high levels of androgens [54]. Elevated circulatory concentrations of OA were correlated with the adverse pregnancy outcomes in obese women with PCOS undergoing controlled ovarian hyperstimulation [14]. Huang et al. 2018 [55] showed that PCOS affects the metabolism of UFAs in rats. In particular, metabolites of AA generated by the cyclooxygenase enzyme were significantly increased in the ovaries of PCOS rats. These AA derived metabolites could modulate the ovarian cycle and induce luteolysis. Niu et al. 2014 [14] showed that levels of OA in the follicular fluid were significantly higher in obese PCOS patients compared to obese women, indicating a role of OA in PCOS. More critical cues on the role of OA can be derived from the regulation of forkhead transcriptional regulator (FOXL2) expression. OA has been shown to have a dose dependent effect on both mRNA and protein expression of FOXL2 in FSH treated cultured bovine GCs [33] (Fig. 2). FOXL2 is vital for the biogenesis of female steroids and is required to prevent the trans-differentiation of the ovary into testis, thus preventing androgen production, as shown in mice [56]. Uhlenhaut et al. 2009 [57] showed that the deletion of FOXL2 immediately increases the expression of testes-specific SRY-box transcription factor 9 (SOX9) in the mouse ovary and as a consequence, leads to comparable testosterone production as in male littermates. In agreement with this earlier report, OA supplementation promoted SOX9 expression in bovine GCs and inhibited E2 biosynthesis [50]. It has been shown that serum anti Mullerian hormone (AMH) levels were increased in patients with PCOS [58]. However, no correlation was observed with respect to circulatory NEFAs and AMH in cows and humans [59, 60]. In any case, future experiments are needed to identify the effects of different NEFAs on AMH production by GCs.

A few reports have shown the effects of UFAs on progesterone (P4) production under serum-supplemented conditions. In goats, OA and LA, but not ALA, could induce P4 production by increasing the phosphorylation of the mitogen-activated protein kinase, ERK1/2 [61]. Supplementation with the ERK1/2 inhibitor U0126 decreased the OA and LA induced P4 production. However, these results indicate that OA and LA may induce premature luteinization of GCs. Zhang et al. 2019 [62] recently reported effects of AA on bovine GCs. It is clear from their data that AA induces ERK and Akt phosphorylation similar to OA and LA. Furthermore, the expression of different marker genes such as *CYP19A1, FSHR, HSD3B1* and *STAR,* and the production of E2 were decreased by AA in a dose-dependent manner. Interestingly, AA dramatically increased the production of P4, which is quite unexpected considering the downregulation of *HSD3B1*. This indicates that the correlation of *HSD3B1* gene expression and P4 progesterone production may not be stringent at the transcriptional level [62]. On the other hand, unlike in goats, OA supplementation did not affect P4 production in bovine GCs cultured with 10% fetal calf serum (FCS) [28]. These opposite effects might be partly attributed to species differences and to the addition of serum in the culture media, whereas GCs inside the ovarian follicle have no direct contact with blood serum due to the non-vascularized GCs layer. It is well known that IGF-1 and FSH signaling plays a vital role in GCs proliferation via the protein kinase A (PKA) and phosphoinositide 3-kinase (PI3K) pathways. It is reported in goats that OA could affect the IGF-1 but not the FSH-induced proliferation of GCs under serum-supplemented conditions.

Overall, unlike for SFAs, it is not completely clear whether UFA metabolites can induce apoptosis. However, multiple reports state that OA, CLA, and AA can down-regulate E2 production and perhaps also the proliferation of GCs. Furthermore, MUFAs could play a role in the etiology of PCOS as they could promote androgen biosynthesis. Studies are yet to be performed to clarify the role of other UFAs such as ALA on GC function.

## Effects of NEFA on oocytes and early embryonic development

During follicular development, oocytes undergo an essential maturation process to become competent for fertilization. Therefore, the composition of biomolecules in the follicular microenvironment is a critical factor determining the fate of the oocyte. Several researchers have studied the effects of NEFAs on oocyte maturation and developmental competence to understand the plausible role of NEFAs in metabolic stress-induced subfertility [17, 29, 44, 48, 63, 64]. Aardema et al. 2011 [48] inferred that NEFAs must be transferred through the cumulus cell layers to reach the oocyte. The transmembrane fatty acid translocase, CD36 mediates the uptake of NEFA in cumulus cells from the follicular fluid. The transport of FAs from cumulus cells to oocytes was thought to take place via gap junctions with the help of FA binding proteins [65]. However, FA transport into the oocyte is yet to be fully understood. Upon entering the oocyte, NEFAs are involved in the generation of lipid droplets (LDs), which are dispersed in the cytoplasm of the oocyte. LDs in oocytes may play a key role in oocyte homeostasis, as they were found to be functionally associated with the mitochondria, ER, endosomes, peroxisomes, and cytoskeleton [66, 67]. During the subsequent developmental progression of the oocyte, most LDs are degraded, and the liberated NEFAs are utilized for essential cellular functions, including mitochondrial oxidation in the embryo [68]. Therefore, LD accumulation has been considered as a valid marker for healthy oocyte maturation in bovine and human in vitro fertilization (IVF) procedures [69]. However, elevated levels of NEFAs in the follicular fluid have been widely reported to cause poor COC morphology, affect oocyte maturation and decrease the numbers of cleaved embryos in humans, cows, pigs, and mice [17, 63, 64, 70, 71]. These adverse effects can also be witnessed at the DNA level as embryos generated under high NEFA exposure have altered DNA methylation and transcriptomic fingerprints of genes related to cell death and metabolic disorder [72]. Following fertilization, the presumed zygote undergoes extensive chromatin remodeling, which includes DNA methylation [73]. DNA (cytosine-5)-methyltransferase 3A (*DNMT3A*) plays a vital role during both the growth and differentiation of mammalian oocytes, especially during maturation and early development [74]. High NEFA exposure downregulates the expression of *DNMT3A*, indicating a direct link between NEFAs and epigenetic programming of embryos [63]. Therefore, it appears that metabolic stress induced subfertility could also be due to epigenetic programming induced by NEFAs in the embryo. In the following sections, we review the major effects induced in oocytes by SFAs and UFAs in different species.

### Saturated fatty acids

**Fig. 3:**
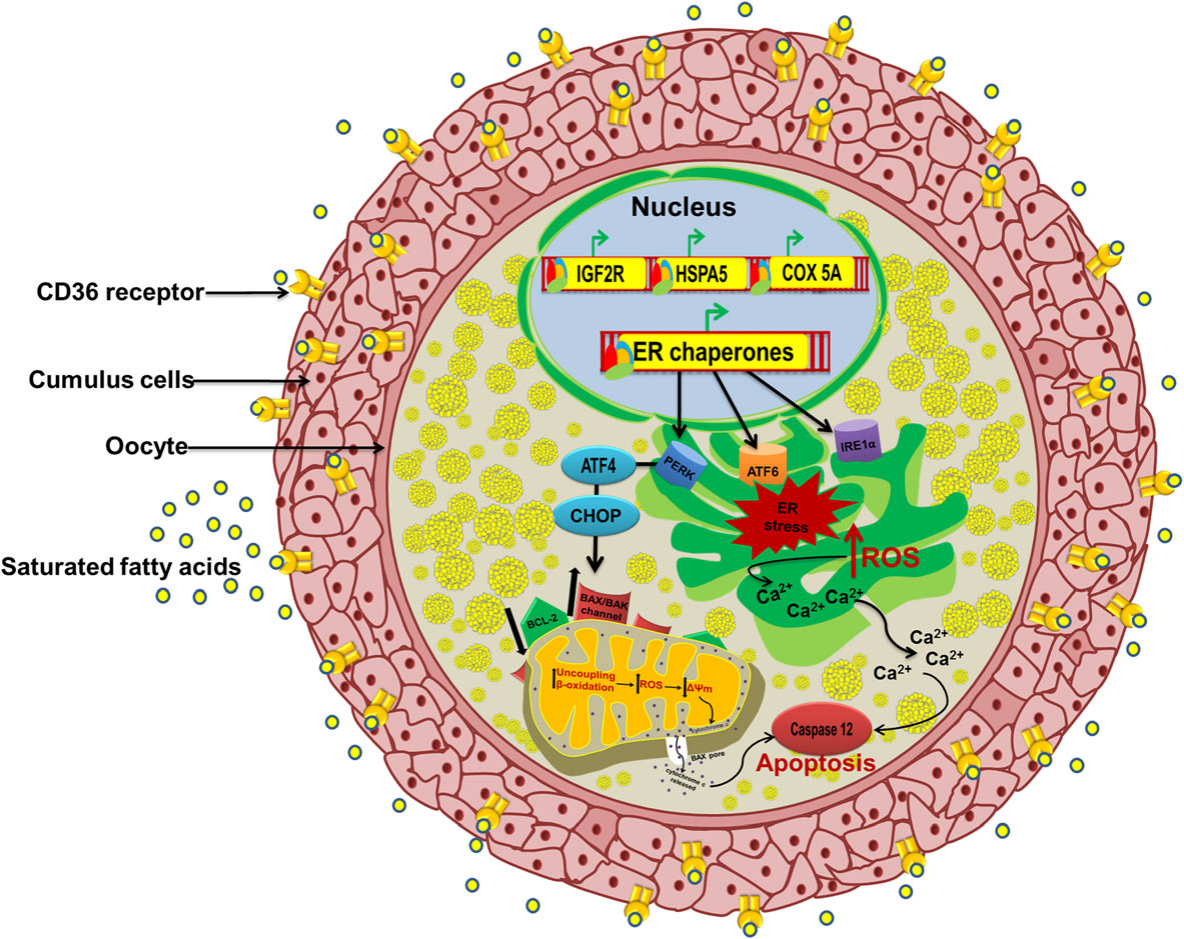
Effects of elevated levels of saturated FAs on oocytes: Elevated levels of saturated fatty acids induce ER stress due to the accumulation of misfolded proteins in the ER lumen, which induces the ER transmembrane signaling proteins PERK, IRE-1 and ATF-6. Induction of C/EBP homology protein (CHOP) via ATF-4 produces excessive reactive oxygen species (ROS) which leads to impaired mitochondrial membrane potential (ΔΨm) and to the release of stored intracellular calcium ions from the ER lumen into the cytosol, eventually initiating a downstream apoptotic cascade. All these events lead to impaired oocyte maturation and developmental competence

A growing number of publications report that SFAs have detrimental effects on oocyte quality. A recent analysis in bovine oocytes revealed that blastocysts derived after PA treatment (150 μM) were inferior in quality and had a high proportion of apoptotic cells in the inner cell mass [75]. This observation is in line with an earlier report showing that exposing bovine oocytes to high concentrations of SA resulted in a significant reduction of the number of oocytes that reach the blastocyst stage with high expression of the maternally imprinted gene *IGF2R* [64] *(*Fig. *3**). D*ysregulation of *IGF2R* perturbs placental and fetal growth [76]. Supplementing of the in vitro maturation medium of COCs with SFAs inhibits the cumulus expansion and progression of oocytes from metaphase II to blastocysts in bovines [17]. Later studies revealed that these FAs could further reduce the survival of bovine blastocysts under in vitro conditions [77]. Similar adverse effects of SFAs have been documented in ewes, where oocytes matured in the presence of 60 μM PA showed signs of impaired maturation, decreased viability, cleavage, and embryo production rates [78]. In contrast, Sinclair et al. 2008 [79] showed that increased levels of SA in the follicular fluid have been associated with the generation of morphologically favorable COCs, but increasing PA levels were associated with poor COCs bovines.

SFAs induce apoptosis by inducing mitochondrial dysfunction and ER stress in maturing oocytes [43]. PA is reported to induce significant alterations in the expression of genes related to the mitochondrial activity (cytochrome c oxidase subunit 5a (*COX5A*)) and oxidative stress (calreticulin, heat shock protein 90 kDa beta member 1 (*HSP90B1*)) of COCs, which is followed by a reduced rate of cleavage and quality of embryos 48 h post-insemination [75, 80]. Similarly, increased concentrations of SFAs facilitate the production of apoptotic inducers such as ceramide and reactive oxygen species (ROS) together with the altered mitochondrial membrane potential [81, 82], which can negatively affect oocyte quality and embryo development (Fig. 3). Increased ROS production is associated with frequent Ca^2+^ oscillations from the ER lumen into the cytosol, as reported in H_2_O_2_ treated mouse oocytes, thus promoting apoptosis [83, 84]. An increased intracellular free Ca^2+^ concentration leads to the activation of Ca^2+^-dependent proteases micro-calpain and caspase-12 in bovine preimplantation embryos [85].

Apart from altered calcium signaling, the expression of classic ER Stress markers such sas *ATF4* (activating transcription factor 4) and *HSPA5* (heat shock protein 5) is also upregulated in COCs matured in the presence of NEFA mixtures containing mainly SFAs, suggesting that protein misfolding pathways can also be disrupted by high NEFAs levels [43] (Fig. 3). Similar expression patterns of ER stress markers were reported in situ and in vitro in mouse COCs exposed to lipid-rich environments, and also in COCs of obese women [26, 86, 87]. ER stress also leads to the accumulation of misfolded proteins, releasing the ER chaperone,78-kDa glucose-regulated protein (GRP78), which leads to the aggregation of ER transmembrane signaling proteins mainly PERK (*protein* kinase RNA-like endoplasmic reticulum kinase), IRE1 (inositol-requiring enzyme *1)* and ATF6 (activating transcription factor 6) and thus commencing the UPR (unfolded protein response) [88]. PERK, C/EBP homologous *protein* (CHOP), and ATF4 (activating transcription factor 4) are functionally associated with each other and induce translocation of the pro-apoptotic protein BAX from the cytosol to the mitochondria and reduce the anti-apoptotic protein BCL-2 [89, 90]. Therefore, chronic ROS generation, excessive Ca^2+^ release, and high expression of proapoptotic proteins can lead to the formation of the BAX pore followed by the release of cytochrome-c and the activation of the caspase cascade in mammalian oocytes [91]. Interestingly, the lipotoxicity induced by SFAs appears to be different between the oocyte and cumulus cells, as revealed by proteomic analysis in which only 4 out of 136 regulated proteins were found to be commonly regulated between the bovine cumulus cells and oocytes [75]. However, the majority of regulated proteins were located in the ER and mitochondria in both groups, indicating the significance of the unfolded protein response and apoptosis upon exposure to SFAs.

Overall, a majority of earlier studies are in agreement that SFAs induce ER stress and disrupt mitochondrial function, thus causing impaired developmental competence in oocytes of different species. However, there is a lack of consensus over the effects of SA on oocyte maturation and health, which can be clarified by future research.

### Unsaturated fatty acids

Accumulating scientific data show that the nutritional supplementation of unsaturated fats could benefit female reproduction by improving the ovarian follicular development and oocyte developmental competence. In particular, PUFAs enriched diets are known to alleviate the NEB, which is a major detriment to female fertility and thus for the herd development in cattle and buffalo during early lactation [92]. Robinson et al. 2002 [93] reported that the size of the dominant follicle increased in cows fed LA enriched diets, whereas cows fed ALA-rich diets showed increased levels of E2 during the follicular phase. Further studies revealed that PUFAs could also improve oocyte developmental competence as the number of blastomeres in morulae increased in cows fed with flaxseed (rich in ALA) compared to those fed with saturated fat (high in PA and SA) or sunflower seed (high in LA). The above nutritional data indicate that UFAs supplementation, especially PUFAs supplementation, could help to improve oocyte development.

However, there are uncertainties in asserting the effects of other UFAs on oocyte function as several in vitro reports emphasize discrepancies in different types of FAs and their corresponding effects on oocyte competence. Jorritsma et al.2004) [28] showed that treatment with UFAs such as OA delayed the maturation of COCs, decreased the fertilization and embryo cleavage rates, and had an overall negative impact on embryonic development. Supplementation with a combination of OA together with PA and SA, resulted in a similar outcome with a reduced number of oocytes reaching the blastocyst stage [64]. In contrast, Aardema et al. 2013 [7] showed that OA is quite harmless to oocytes even at higher concentrations. Another UFA, LA, has also been reported having adverse effects on bovine oocyte development. Increased concentrations of LA dramatically reduced cumulus expansion by decreasing the phosphorylation of Akt and mitogen-activated protein kinase-3 (MAPK3). LA supplementation also reduced the cyclic adenosine monophosphate (cAMP) production in bovine COCs [94]. In a subsequent paper, Marei et al. 2012 [95] reported that LA could alter the mitochondrial distribution, decrease the mitochondrial membrane potential, and increase ROS production in bovine oocytes undergoing maturation. However, these negative effects of LA were counterbalanced by the supplementation of antioxidants, such as vitamin E and glutathione peroxidase [96]. On the other hand, ALA has no such negative effects on oocyte function at physiological concentrations. Interestingly, Marei et al. 2017 [97] reported that ALA could protect bovine oocytes against the combined lipotoxic effects of elevated PA, SA and OA by improving their developmental competence through increased mitochondrial activity and reduced ER stress levels and apoptosis in bovine cumulus cells.

It has been shown that stearoyl Co-A desaturase (SCD) activity is closely associated with lipid metabolism in dairy cows and may influence their reproductive performance [98]. SCD catalyzes the conversion of SA and PA into oleate and palmitoleate, respectively. Increased expression of stearoyl-CoA desaturase-1 (SCD1) and stearoyl-CoA desaturase-5 (SCD5) in human and SCD1 in bovine cumulus cells was correlated with improved oocyte competence [99, 100]. Importantly, SCD activity is inhibited by SA in bovine cumulus cells of intact COCs, thus causing impaired oocyte development compared to denuded oocytes exposed to SA in the absence of cumulus cells [100]. SCD inhibition in human COCs not only leads to compromised oocyte development but also to reduced aromatase expression, which is followed by decreased E2 production [101].

It appears that UFAs, particularly, OA and ALA can reduce the lipotoxicity induced by SFAs during oocyte maturation and blastocyst formation. However, OA and LA also have detrimental effects on oocyte maturation and developmental competence. No adverse impacts of ALA have been documented, and perhaps supplementing omega 3 PUFAs in the diet and during in vitro culture conditions could be highly beneficial to the oocyte function in comparison to other UFAs.

## Concluding remarks

A variety of metabolic conditions/diseases are characterized by elevated concentrations of NEFAs in the blood and eventually in the follicular fluid of humans, cows, sheep, and pigs. Based on the reviewed scientific data, it is apparent that the degree of unsaturation in the acyl carbon chain of fatty acids could determine their biological effects on GCs and oocytes. Both normal and elevated levels of SFAs, and elevated levels of MUFAs (e.g. OA) and PUFAs (e.g. LA and AA) have severe detrimental effects on the GC function and developmental competence of oocytes, thus acting as foes in the ovarian follicle. No negative impacts of omega-3 fatty acids (ALAs) have been reported on GCs and oocytes, and perhaps they can be considered as friends in the ovarian follicle. Given the essential nature of FAs, such as LA and ALA, we think that metabolic diseases may not raise the concentration of LA and ALA in the circulation. Therefore, the levels of PA, SA and OA are the major detriments to ovarian function during metabolic diseases. The combined lipotoxicity of these three FAs can be ameliorated by ALA in GCs and oocytes. More studies are yet to be performed with respect to their intracellular signaling, especially PKA, Akt, and mitogen-activated protein kinase (MAPK) pathways, which would help to better understand their effects in cross-talk with FSH and IGF1 signalling and could help to generate novel therapeutics for subfertility.

## Data Availability

Data sharing is not applicable to this article as no datasets were generated or analysed during the current study.

## Abbreviations

AA: Arachidonic acid
ACO: Acyl CoA oxidase
ALA: α-linolenic acid
AMPK: 5′ adenosine monophosphate-activated protein kinase
ATF4: Activating transcription factor 4
cAMP: Cyclic adenosine monophosphate
CCND2: Cyclin D2
CLA: Conjugated linoleic acid
COC: Cumulus oocyte complex
CPT1: Carnitine palmitoyl transferase-1
CYP11A1: Cytochrome P450 family 11 subfamily A member 1
CYP19A1: Cytochrome P450, family 19, subfamily A, polypeptide 1
E2: 17β-estradiol
ESR2: Estrogen receptor 2
FA: Fatty acid
FOXL2: Forkhead box protein L2
FSH: Follicle-stimulating hormone
FSHR: Follicle-stimulating hormone receptor
FST: Follistatin
GC: Granulosa cells
GnRH: Gonadotropin-releasing hormone
HDL: High-density lipoproteins
HSD3B1: 3beta-hydroxysteroid dehydrogenase/delta (5)-delta (4) isomerase type I
HSPA5: Heat shock protein family A (Hsp70) member 5
IGF-1: Insulin-like growth factor 1
LA: Linoleic acid
LDL: Low-density lipoproteins
LH: Luteinizing hormone
MAPK: Mitogen-activated protein kinase
MUFA: Monounsaturated fatty acid
NEB: Negative energy balance
NEFA: Non-esterified fatty acids
OA: Oleic acid
PA: Palmitic acid
PCNA: Proliferation cell nuclear antigen
PCOS: Polycystic ovarian syndrome
PI3K: Phosphoinositide 3-kinase-3-kinase
PKA: Protein kinase A
PPAR: Peroxisomal proliferator-activated receptors
PUFA: Polyunsaturated fatty acid
SA: Stearic acid
SCD: Stearoyl coA desaturase 1
SMASE: Sphingomyelinase
SOX9: SRY (sex determining region Y)-box 9
SPT: Serine palmitoyltransferase
VLDL: Very low-density lipoproteins
